# Polyamines: Potential anti-inflammatory agents and their possible mechanism of action

**DOI:** 10.4103/0253-7613.42305

**Published:** 2008-06

**Authors:** Chakradhar V. Lagishetty, Suresh Ramnath Naik

**Affiliations:** Department of Pharmacology and Toxicology, Prin. K. M. Kundnani College of Pharmacy, Jote Joy Building, Rambhau Salgaonkar Marg, Cuffe Parade, Mumbai - 400 005, India

**Keywords:** Arthritis, carrageenin paw edema, cotton pellet granuloma, lipid peroxides and lysosomal enzymes, polyamines

## Abstract

**Objective:**

To evaluate the anti-inflammatory activity of exogenously administered polyamines on experimentally induced acute and chronic inflammation in wistar rats and to elucidate their possible mechanism of action.

**Materials and Methods:**

The *in vivo* anti-inflammatory activity of polyamines was studied using acute (carrageenin paw edema), sub-acute (cotton pellet granuloma) and chronic (Freund's adjuvant induced arthritis) models of inflammation. The biochemical parameters like liver lipid peroxides, SGOT and SGPT were also measured.

**Results:**

Polyamines exhibited significant anti-inflammatory activity in acute, sub-acute and chronic models of inflammation. Polyamines treatment inhibited the increase in lipid peroxides in liver and the serum concentration of marker enzymes (glutamate oxaloacetate transferase and glutamate pyruvate transferase) during inflammation.

**Conclusion:**

Polyamines possess anti-inflammatory activity in acute and chronic inflammation which can be attributed to their anti-oxidant and /or lysosomal stabilization properties.

## Introduction

The polyamines putrescine, spermidine and spermine are aliphatic polycations derived from ornithine and play vital physiological roles.[1] Polyamines being cationic in nature bind to the negatively charged nucleic acids of the cell membrane surface and are thus involved in stabilization of the membrane structures.[2] Polyamines are involved in cellular processes such as DNA and protein synthesis. They participate in cell proliferation and differentiation.[3] They also act as scavengers of reactive oxygen species and thereby protect DNA, proteins and lipids from oxidative damage.[4]

Polyamines have been postulated to have anti-inflammatory and anti-oxidant properties.[1] It has been suggested that they exert at least two different anti-inflammatory mechanisms, the first one is mediated by the synthesis of an anti-inflammatory protein (vasoregulin)[5] and the second one is their direct action on leucocytes.[6] Most of the reported studies however have been conducted on several cell-lines *in vitro* and those *in vivo* have been of a preliminary nature. Also, they have not been investigated in chronic models of inflammation. The present study was undertaken with an objective to evaluate the anti-inflammatory activity of polyamines in different models of acute, subacute and chronic biochemical parameters. Several have also been investigated to correlate them with the anti-inflammatory activity of polyamines.

## Materials and Methods

### Animals

Wistar strain rats (120-150 g) were purchased from the registered breeder Bharath Sera Pvt Ltd., Mumbai, India. The rats were housed hygienically under standard conditions of temperature (24 ± 1°C), relative humidity (65 ± 10%) and light (10h) dark (14h) cycle. They were fed with standard pellet food (Amrut Laboratory animal feed diet, Maharashtra, India) and water *ad lib*.

### Drugs, chemicals and reagents

Freund's adjuvant (complete) was a kind gift sample from Bharat Sera Vaccines, India. Carrageenin was purchased from Sigma-Aldrich, USA. Kits for SGOT and SGPT determination were purchased from Span diagnostics, Gujarat, India. All other chemicals used were of analytical reagent grade and were procured from local suppliers.

### Animal models

Institutional animal ethics committee approval

All experimental protocols were approved by the institutional ethics committee.

Acute inflammation - Carrageenin induced hind paw edema in rats: Edema was produced acutely by injecting subcutaneously (s.c) 0.1 mL of carrageenin (1% w/v), into the plantar region of the hind paws of the rat according to the method of Winter *et al.*[7] Male wistar rats weighing between 120-150 g were used. A mark was made on both hind paws just below the tibiotarsal junction so that the paw could be dipped in the mercury column of the plethysmometer upto the mark to ensure constant paw volume. Putrescine (100 mg/kg), spermidine (17.5 mg/kg), spermine (12.5 mg/kg) and mixture of polyamines (100 mg/kg of putrescine + 17.5 mg/kg of spermidine + 12.5 mg/kg of spermine) were administered s.c in the neck region 3h prior to carrageenin injection[5] and paw volumes were measured at 2, 4 and 6 h after carrageenin injection. A control group treated with the vehicle (0.1 mL saline per 100 g) was used for comparison. Animals were sacrificed under light ether anesthesia at the end of 6 h, blood was collected by cardiac puncture for biochemical determinations like SGOT and SGPT by the method of Reitman and Frankel[8] and lipid peroxides in liver homogenates by the method of Ohkawa *et al.*[9] Paw edema was calculated for each rat by subtracting the baseline reading from that of 2, 4 and 6 hour. The anti-inflammatory activity of polyamines was determined by the following formula:

$$\text{\% inhibition of edema}=\left[1-\frac{\text{Mean increase in paw volume in Polyamines treated group of rats}}{\text{Mean increase in paw volume in saline treated group of rats}}\right]\times 100$$

2.3.2 Sub-acute inflammation - Cotton pellet granuloma in rats: The cotton pellet granuloma was produced in rats by the method of Winter and Porter with slight modification.[10] The pellets, weighing exactly 10 mg each, were made from 5 mm sections of cotton rolls. The cotton pellets were sterilized in an autoclave for 30 minutes at 120°C under 15 lb pressure. Four pellets were inserted s.c into the ventral region, two on either side, in each rat under light ether anesthesia. Vehicle (0.1mL saline per 100 g), putrescine (25 mg/kg), spermidine (7 mg/kg), spermine (5 mg/kg) and mixture of polyamines (25 mg/kg of putrescine + 7 mg/kg of spermidine + 5 mg/kg of spermine) were administered daily for 7 days subcutaneously in the neck region. Animals were sacrificed on the 8^th^ day, blood was collected by cardiac puncture for SGOT and SGPT determination. The liver was separated and lipid peroxides in liver homogenate were determined. The cotton pellets (along with the granular tissue formed around) were removed surgically and freed from extraneous tissue. The pellets were weighed immediately for wet weight. Then, pellets were dried in an incubator at 60°C until a constant weight was obtained (all the exudate dried up). The exudate amount (weight of exudate in mg) was calculated by subtracting the constant dry weight of pellet from the immediate wet weight of pellet. The granulation tissue formation (dry weight of granuloma) was calculated after deducting the weight of cotton pellet (10 mg) from the constant dry weight of pellet and taken as a measure of granuloma tissue formation. The percent inhibitions of exudate and granuloma tissue formation were determined as follows:

$$\text{Exudate inhibition (\%)}=\left[1-\frac{\text{Weight of exudate in mg of polyamine treated group of rats}}{\text{Weight of exudate in mg of saline treated group of rats}}\right]\times 100$$

$$\text{Granuloma inhibition (\%)}=\left[1-\frac{\text{Weight of granuloma in mg of polyamine treated group of rats}}{\text{Weight of granuloma in mg of saline treated group of rats}}\right]\times 100$$

### Chronic inflammation

Freund's adjuvant induced arthritis: Freund's adjuvant (0.1mL, complete) was injected s.c into the plantar region of the right hind paw of the rat according to the method described by Stoerk *et al*[11] and Weichman.[12] Paw volume was measured on every alternative day for 21 days using volume displacement plethysmometer. Vehicle (0.1 mL saline per 100 g rat), putrescine (12.5 mg/kg), spermidine (3.5 mg/kg), spermine (2.5 mg/kg) and mixture of polyamines (12.5 mg/kg of putrescine + 3.5 mg/kg of spermidine + 2.5 mg/kg of spermine) were administered s.c in the neck region from the day of adjuvant injection daily for 14 days and animals were observed for arthritic symptoms like primary lesion (injected paw), secondary lesions (non-injected paw swelling), knee joint movements and pain threshold (assessed by vocal reflex – squeaks), grip strength (assessed by holding capacity on a wire mesh inclined plane when varying weights are attached to its tail) and paw volume were measured on every alternative day for 21 days. The animals were sacrificed on 22^nd^ day and blood was collected by cardiac puncture for the determinations of SGOT, SGPT and the lipid peroxides in liver homogenate were also measured.

The quantitative estimation of lipid peroxidation was done by determining the concentration of thiobarbituric acid reactive substance (TBARS) in the liver using the method of Ohakawa *et al.*[9] The amount of malondialdehyde (MDA) formed was quantified by reaction with TBA and used as an index of lipid peroxidation. The results were expressed as nanomoles of MDA/g of wet liver using molar extinction coefficient of the chromophore (1.56 × 10^−5^ M/cm).

### Statistical analysis

One-way ANOVA with Dunnett's post test was performed using GraphPad InStat version 3.00 for Windows 95 (Graphpad Software, San Diego California USA)

## Results

Individual polyamines elicited a significant anti-inflammatory activity in carrageenin edema test. However, a mixture of the three polyamines did not elicit a synergistic activity [Table 1].

**Table 1:** Effect of exogenously administered polyamines on rat carrageenin edema

	*Mean volume of edema (ml) ± SEM*				
	
*Time after carrageenin injection (h)*	*Saline treated*	*Putrescine (100 mg/kg)*	*Spermidine (17.5 mg/kg)*	*Spermine (12.5 mg/kg)*	*Mixture*#
2	0.66 ± 0.048 (---)	0.44 ± 0.043 (33)	0.39 ± 0.11* (41)	0.55 ± 0.00 (17)	0.52 ± 0.001 (21)
4	0.77 ± 0.049 (---)	0.42 ± ?0.049* (45)	0.44 ± ?0.11* (43)	0.47 ± ?0.040* (39)	0.43 ± ?0.058* (44)

Figures in parentheses in dicate the % inhibition

* *P*<0.05

**P<0.01 as compared to Saline treated group (one-way ANOVA). N=5/group.

# 100mg/kg Putrescine + 17.5 mg/kg spermidine + 12.5 mg/kg spermine

**Figure 1:**
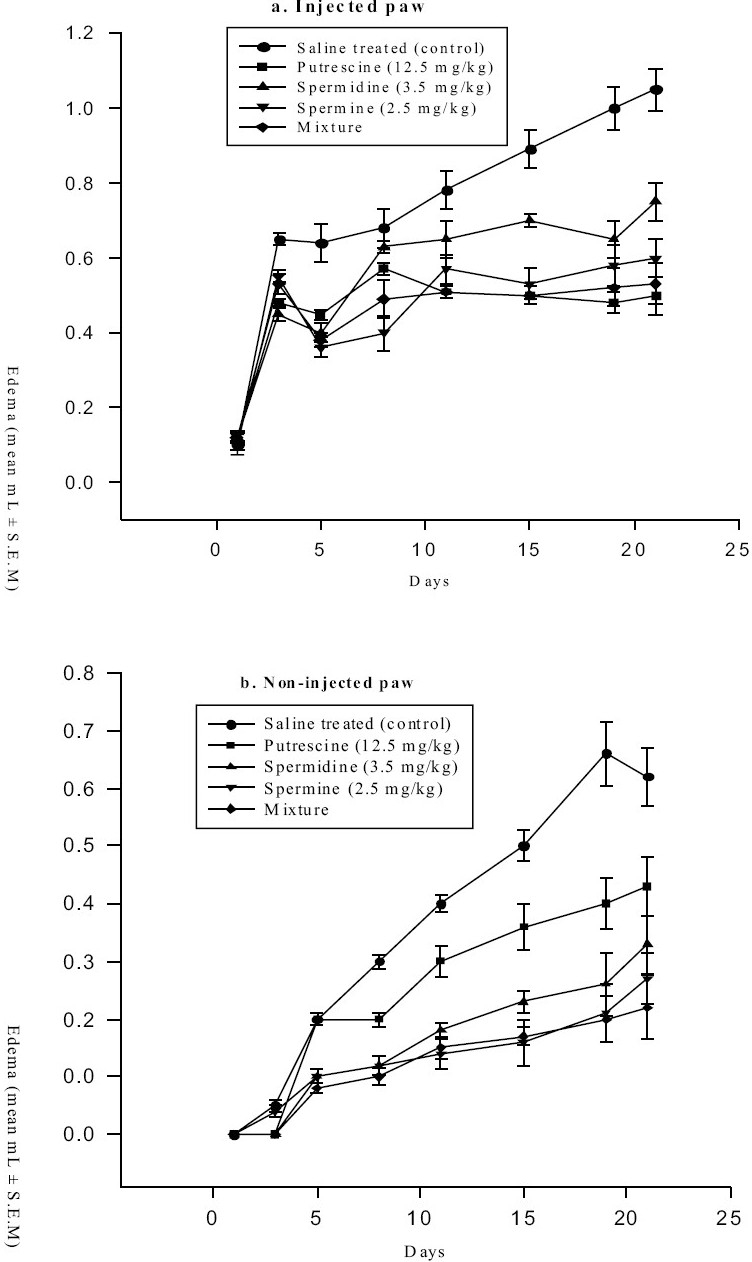
Effect of polyamines on Freund's adjuvant induced arthritis in rats: (a) Injected paw (b) Non-injected paw; N = 5/group

Rats treated with individual polyamines showed a significant inhibition of granuloma tissue as well as exudate formation. Among them, spermine showed the maximum inhibition. Treatment with a mixture of three polyamines showed maximum inhibition of granuloma tissue formation as well as that of exudates [Table 2]. Individual polyamine treatment daily for 14 days in arthritic rats inhibited the primary and secondary lesions, decreased the pain threshold perception and improved the joint movement and grip function during 21 days observation period [Table 3]. However, individual polyamines exhibited varied degree of edema inhibition in the injected and non-injected paws [Figure 1].

**Table 2:** Effect of polyamines on exudation and granular tissue formation in cotton pellet granuloma in rats

*Treatment (Subcutaneous does*	*Weight of exudate (mg)*	*Exudate inhibition (%)*	*Dry weight of granuloma (mg)*	*Granuloma inhibition (%)*
Saline (0.1ml/100 g rat)	101.23 ± 3.28	-	25.86 ± 0.92	-
Putrescine (25 mg/kg)	85.25 ± 3.89	15.79	19.05 ± 1.44	26.33
Spermidine (7 mg/kg)	82.95 ± 3.92	18.06	19.60 ± 1.35	24.21
Spermine (5 mg/kg)	75.92 ± 4.24	25.00	17.26 ± 1.47	33.26
Mixture (25 mg/kg putrescine	74.64 ± 3.22	26.27	17.20 ± 1.26	33.49
+ 7 mg/kg spermidine				
+ 5 mg/kg spermidine)				

Values are expressed as Mean ± S.E.M.; N = 5/group. *P*<0.05; *P*<0.01 as compared to saline treated (one-way ANOVA)

**Table 3:** Effect of polyamine treatment on Freund's adjuvant induced arthritis

*Treatment (Subcutaneous dose)*	*Primary lesions*	*Secondary lesions*	*Degree of pain threshold*	*Degree of joint movements*	*Degree of grip function*
Control (saline 0.1 ml/100 g rat)	++++	+++	++++	++++	++++
Putrescine (12.5 mg/kg)	+	++	++	++	++
Spermidine (3.5 mg/kg)	++	+	++	++	++
Spermine (2.5 mg/kg)	+	+	++	++	++
Mixture (putrescine 12.5 mg/kg	+	+	++	++	++
+ spermidine 3.5 mg/kg					
+ spermine 2.5 mg/kg)					

++++ Maximum/severe; +++ Moderate; ++ Mild; + Nil/very mild; N=5/group

The treatment with polyamines individually as well as together (mixture) inhibited significantly the increase in SGOT and SGPT levels in different types of experimental inflammation. The MDA content of the liver homogenate was increased significantly in all types of experimental inflammation as compared to normal group. Treatment with polyamines individually as well as together (mixture) significantly reversed the elevated lipid peroxides. The degree of reversal was slightly more with a mixture of polyamines. All individual polyamines reversed the increase in lipid peroxide formation in all types of inflammation [Table 4].

**Table 4:** Effect of Polyamine treatment on SGOT, SGPT and lipid peroxidation during carrageenin edema, cotton pellet granuloma and Freund's adjuvant induced arthritis

*Animal model*	*Treatment (Subcutaneous dose)*	*SGOT (Units/ml)*	*SGPT (Units/ml)*	*Lipid peroxides (nmol of MDA g^−1^ tissue)*
Carrageenin edema	Saline (0 mg/kg)	247.89±8.06	75.54±3.26	27.84±0.34
	Putrescine (100 mg/kg)	213.16±3.92	58.82±2.25	24.34±0.26
	Spermidine (17.5 mg/kg)	217.53±10.59	46.18±1.35	14.39±0.89
	Spermine (12.5 mg/kg)	199.34±3.91	63.15±4.18	21.02±1.39
	Mixture^a^	202.80±2.80	48.14±1.42	18.32±1.12
Cotton pellet granuloma	Saline (0 mg/kg)	121.09±7.36	71.03±2.50	27.03±0.34
	Putrescine (25 mg/kg)	93.85±5.86	53.68±3.87	20.04±1.50
	Spermidine (7 mg/kg)	95.83±3.83	50.45±5.93	12.51±1.20
	Spermine (5 mg/kg)	78.20±5.19	58.30±4.16	14.66±1.08
	Mixture^b^	76.40±4.82	50.90±5.12	13.64±1.42
Freund's adjuvant induced arthritis	Saline (0 mg/kg)	162.04±3.68	62.56±1.35	23.67±0.79
	Putrescine (12.5 mg/kg)	150.98±4.72	45.09±3.92	19.10±0.35
	Spermidine (3.5 mg/kg)	148.86±7.19	48.20±2.16	18.02±0.35
	Spermine (2.5 mg/kg)	142.36±5.08	43.65±3.68	19.37±0.22
	Mixture^c^	152.34±6.58	44.06±3.21	19.25±0.36

Values are expressed as Mean±S.E.M. *P*<0.05 and *P*<0.01 as compared to saline treated group. N = 5/group.

a 100 mg/kg Putrescine + 17.5 mg/kg spermidine + 12.5 mg/kg of spermine

b 25 mg/kg putrescine + 7 mg/kg spermidine + 5 mg/kg spermine

c putrescine 12.5 mg/kg + spermidine 3.5 mg/kg + spermine 2.5 mg/kg.

## Discussion

The previous studies have indicated elevated polyamines levels in edematous and granulomatous tissue during acute and sub chronic experimental models of inflammation.[13] The increased polyamines levels are known to trigger negative immune regulators by their action on lymphocytes, neutrophil locomotion and natural killer cell activity.[14–16] Furthermore, the involvement of spermine in macrophage cytokine synthesis inhibition has also been reported.[17] These experimental findings clearly suggest that endogenous polyamines act as regulators of the inflammatory process. Our efforts were directed to observe the effect of exogenous polyamines on different types of experimental inflammation.

Our experiments showed that exogenous polyamines treatment either individually or as a mixture elicited a significant anti-inflammatory activity in carrageenin (acute), cotton pellet granuloma (sub-acute) and freunds-adjuvant polyarthritis rat models. Furthermore, polyamine treatment prevented the increase in lysosomal marker enzymes SGOT, SGPT and increased liver peroxides during different type of inflammatory conditions significantly. The inhibition of lysosomal marker enzymes may be largely due to membrane stabilizing property of polyamines.[18] Polyamines are known to exhibit antioxidant activity[19–21] and the same has been confirmed in our experiments too. It is likely that both antioxidant activity as well as a membrane stabilizing effect of polyamines might be contributing to their anti-inflammatory activity observed in different types of inflammation. The other plausible explanation may be due to a negative immune regulation via their effect on lymphocytes and/or neutrophil locomotion or natural cell killer activity. Furthermore, the direct involvement of polyamines in stimulation of the synthesis of vasoregulin, a known anti inflammatory protein by its direct action on leukocytes had been reported.[22] This inherent effect of polyamines cannot be ignored for the observed anti-inflammatory activity of polyamines.

In conclusion, our experimental results suggest that exogenous polyamines administered by subcutaneous route exhibit anti-inflammatory activity in acute and chronic inflammation. A combined treatment with three polyamines exhibits no synergistic activity. The possible mechanism of anti-inflammatory activity of polyamines may be due to their inherent anti-oxidant activity and/or lysosomal membrane stabilization as supported by our experimental findings. Thus, studies on polyamines may be helpful in developing a new approach for better understanding of the inflammatory process and the generation of new anti-inflammatory drugs.
